# Factorial Optimization of Ultrasound-Assisted Extraction of Phycocyanin from *Synechocystis salina*: Towards a Biorefinery Approach

**DOI:** 10.3390/life12091389

**Published:** 2022-09-06

**Authors:** Joana Assunção, Helena M. Amaro, Francisco Xavier Malcata, Ana Catarina Guedes

**Affiliations:** 1 CIIMAR/CIMAR-LA—Interdisciplinary Centre of Marine and Environmental Research, Novo Edíficio do Terminal de Cruzeiros do Porto de Leixões, Av. General Norton de Matos, s/n, 4450-208 Matosinhos, Portugal; 2 LEPABE—Laboratory for Process Engineering, Environment, Biotechnology and Energy, Faculty of Engineering, University of Porto, Rua Dr. Roberto Frias, s/n, 4200-465 Porto, Portugal; 3 ALiCE—Associate Laboratory in Chemical Engineering, Faculty of Engineering, University of Porto, Rua Dr. Roberto Frias, 4200-465 Porto, Portugal; 4 FEUP—Department of Chemical Engineering, Faculty of Engineering, University of Porto, Rua Dr. Roberto Frias, s/n, 4200-465 Porto, Portugal

**Keywords:** cyanobacteria, bioactive pigments, successive extraction, phycocyanin, optimization, Box–Behnken model, biorefinery

## Abstract

PC is a bioactive and colorant compound widely sought in the food, nutraceutical and cosmetic industries, and one of the most important pigments produced by *Synechocystis salina*. However, the general extraction process is usually time-consuming and expensive, with low extraction yields—thus compromising a feasible and sustainable bioprocess. Hence, new extraction technologies (e.g., ultrasound assisted-extraction or UAE) emerged in the latest years may serve as a key step to make the overall bioprocess more competitive. Therefore, this study aimed at optimizing the yields of phycocyanin (PC) rich-extracts of *S. salina* by resorting to UAE; in attempts to explore this process in a more economically feasible way; valorization of the remaining cyanobacterial biomass, via extraction of other bioactive pigments and antioxidants, was tackled within a biorefinery perspective. A two-stage extraction (using ethanol and water) was thus performed (because it favors PC extraction); other bioactive pigments, including chlorophyll *a* (chl *a*), carotenoids, and other phycobiliproteins (PBPs), but also antioxidant (AOX) capacity and extraction yields were also evaluated for their optimum UAE yields. A factorial design based on Box–Behnken model was developed; and the influence of such extraction parameters as biomass to solvent ratio (B/S ratio = 1.5–8.5 mg·mL^−1^), duty cycle (DT = 40–100%), and percentage of amplitude (A = 40–100%) were evaluated. The model predicted higher PC yields with high B/S ratio = 6 mg·mL^−1^, lower DT = 80% and an A = 100%. Classical extraction was compared with UAE under the optimum conditions found; the latter improved PC yields by 12.5% and 47.8%, when compared to freeze-thawing extraction, and bead beater homogenization-based extraction, respectively. UAE successive extractions allowed to valorize other important bioactive compounds than PC, by reusing biomass, supporting a favorable contribution to the economic feasibility of the *S. salina*-based process towards a biorefinery approach.

## 1. Introduction

The valorization of cyanobacterial biomass via extraction of natural bioactive pigments has been gaining relevance at industrial level. In fact, industry is developing efforts to replace synthetic pigments by natural ones, since the demand for the latter has been increasing worldwide. Consumers are more and more aware of the potentially negative effects of chemical synthesis upon one’s health, particularly in the food and cosmetic fields—and this has been driving attention to less processed-products, from sustainable sources, and with reduced environmental fingerprint [1].

Cyanobacteria are a relevant source of natural and biodegradable bioactive pigments, such as chlorophyll *a* (chl *a*), carotenoids, and phycobiliproteins (PBPs)—including phycocyanin (PC), allophycocyanin (APC) and phycoerythrin (PE); they hold indeed extremely appealing bright colors [2,3]. Chl *a*-related molecules, carotenoids and PBS share the unique property of absorbing light thanks to their characteristic conjugated double bond network; however, each possesses distinctive features. Chl-related molecules (λ_max_ = 428 nm; λ_max_ = 665 nm) are natural green pigments that function as primary light-harvesting pigments and are composed by a backbone with a tetrapyrrol ring attached to a central magnesium atom. Because of its chemical and structural features, chl exhibits a number of bioactivities beneficial for human health. On the other hand, carotenoids exhibit unique colors that range from yellow to red; in a photosynthetic context, they appear most often associated to chl *a*, thus helping complement their absorbing wavelengths within λ = 400–500 nm. Composed by a backbone of 40-carbon isoprene units, carotenoids can be divided in two main groups: carotenes and xanthophylls, differing by presence or absence of oxygen as functional group, respectively. Owing to their structural features, such compounds possess good antioxidant properties—so they protect organisms against the oxidative stress caused by scavenging reactive oxygen species (ROS). PBPs are colored (blue or red), water soluble-proteins covalently linked to phycobilins—and commonly found as part of the phycobilisome structure in the photosynthetic membranes of cyanobacteria (besides red algae). They maximize photosynthetic efficiency of incident radiation; but also possess important chemical features exhibiting noteworthy functional properties [4].

Extracts rich in the aforementioned pigments can be used as dyes or for health purposes—owing to their nutritional and therapeutic value [5,6,7]. Such pigments possess antioxidant features—and are accordingly able to scavenge free ROS and reactive nitrogen species (RNS). Both ROS and RNS are associated with several diseases (e.g., cataract, arthritis, cancer) and metabolic disorders. Moreover, cyanobacterial pigments hold notable biological activities, including antitumor, antidiabetic, anti-inflammatory, immunomodulatory, and anti-ageing effects [3,5,8,9]. In some cases, those bioactivities can be enhanced by synergistic effects between several compounds, including PC, APC, and PE, in the extracts. This is why they have found application as functional ingredients in food, nutraceutics, cosmetics, and even feed (e.g., aquaculture, pet), in attempts to promote both human and animal well-being [8,10,11].

The market of compounds that may be obtained from cyanobacteria has been increasing, in particular regarding functional ingredients. For instance, the market for PC was estimated as USD 45 million in 2020, and the global market for PBPs from cyanobacteria (i.e., *Arthrospira platensis*) was estimated in almost USD 60 million, with prospects for doubling by 2028 [12]. Therefore, optimization of extraction and fractionation of cyanobacterial biomass toward recovery of such added-value compounds appears crucial, so as to respond to the expected market demand. Extraction and purification have traditionally entailed major economic bottlenecks, because they are high energy-demanding, and often offer low extraction yields; in some cases, they account for up to 70–80% of the total cost of the cyanobacterial-based final product (depending on the compound at stake and its purity specifications) [13]. An economically feasible and sustainable cyanobacterial bioprocess—able to effectively address demand by the nutraceutical, food, cosmetic, feed, and pharmaceutical markets, urges approaches based on the biorefinery and circular economy concepts—resorting to such greener technologies as biomass maximization and solvent reutilization, choice of certain green and cheaper solvents, and obviously optimization of operating conditions [5,6,7,14]. Until now, there is no information regarding a cyanobacterial biorefinery fulfilling or operating under such criteria at larger scale. The available literature only reports fundamental studies at laboratory or small scale, and mainly cover *Arthrospira platensis* [15,16]; however, the potential of other strains is only now starting to be disclosed—as is the case of *S. salina* [5] and *Cyanobium* sp. [6], under a biorefinery context.

Furthermore, researchers have been focusing on novel extraction techniques for cyanobacterial bioactive metabolite-rich extracts, such as ultrasound-assisted extraction (UAE), microwave assisted extraction (MAE), pulsed electric field (PEF), sub- or supercritical fluid extraction (SFE), subcritical water extraction (SWE), supersonic fluid processing, pressurized liquid extraction (PLE), or enzyme-assisted extraction (EAE) [14,17]; all such techniques are cleaner and less aggressive to the environment than their classical counterparts that resort to toxic organic solvents, which raises disposal and environmental issues and are time consuming (e.g., chloroform:methanol Soxhlet extraction, glass bead milling/high shear homogenization, osmotic heating or shock, freezing-thawing cycle) [2].

Due to the intrinsically high robustness of cyanobacterial cells, a combined methodology of mechanical and chemical extraction is normally preferred for pigment extraction [18]. Mechanical extraction often resorts to bead-milling, high-pressure, glass-bead assisted extraction, and freezing-thawing cycles [2], aimed at disrupting the cells. On the other hand, chemical extraction makes uses of organic or aqueous solvents, to assists in mechanical extraction for a more efficient release of compound(s). Both the nature of solvent used and the B/S ratio strongly impact the extraction yields, and efficiency hinges upon the selected strain and the target-product [2,19].

Several studies have employed UAE to obtain bioactive pigment-enriched extracts, including PC [20,21,22,23,24,25]. This technology takes advantage of microbubble sudden formation and collapse, derived from acoustic cavitation, to create macro-turbulence and high-velocity inter-particle collisions in the liquid solvent, along with high shear forces that distort the cell wall [26]. Such disturbances favor solvent penetration in the biomass and mass transfer, which help in the release of intracellular content [27,28]. The contact surface area between solvent and compound of interest actually increases, and this is a major reason for improved mass transfer [21].

UAE is a clean and safer technique that is solvent-mediated, by using GRAS (Generally Recognized As Safe) solvents (i.e., which follow the green chemistry principle, are not toxic, and are safe for use in industry); it is easy to scale-up—thus allowing larger-scale application thereof. UAE is also promising in terms of energy consumption, since it may drastically reduce energy inputs when compared to traditional methods. In addition, UAE demonstrates benefits as it is based on fully non-toxic wave propagation, which do not contaminate the environment from a mechanism point of view [29]. The level of efficiency of this technique is strongly dependent upon extraction time and number of cycles used (i.e., duty cycle), temperature, solvent properties (e.g., polarity), size of sample, volume of solvent used (i.e., biomass to volume ratio), amplitude percentage, and frequency and ultrasound intensity [19,30,31]. However, UAE has already proven several advantages, namely increased extraction yield of cyanobacterial metabolites and reduced extraction times [19,32].

The marine cyanobacterium *S. salina* was selected for this study owing to its potential biotechnological value [5,33]. Beside its potential for bioremediation processes and production of lipids for diesel generation [33], this species was reported to possess considerable amounts of PC and important antioxidant properties regarding its ethanolic and aqueous extracts [5]. The generation of bioactive extracts from *Synechocystis* represents a major opportunity to improve compound and pigment recovery techniques, and (re)use its biomass in combination with GRAS solvent-mediated extraction (e.g., ethanol, water) towards a greener and feasible process within a biorefinery approach. In view of the above, this study focused on optimization of the UAE-based extraction yields PC aqueous-rich extracts from *S. salina* LEGE 06155, under the general principles of biorefinery. Hence, a two-stage extraction (using ethanol followed by water, as GRAS solvents) was applied towards maximization of PC and other bioactive pigment extraction (chl *a*, carotenoids and other PBPs, including APC and PE). The corresponding extraction yields and antioxidant capacity were also evaluated. A factorial design was applied by resorting to Box–Behnken design, after selection of B/S ratio (1.5–8.5 mg·mL^−1^), duty cycle (40–100%), and percentage of amplitude (40–100%) for extraction parameters. For UAE model comparison, conventional extractions resorting to heat-solvent agitation (to obtain ethanolic extracts) followed by freeze-thawing (to obtain aqueous extracts) were performed in parallel; as well as homogenization by bead beating.

## 2. Materials and Methods

### 2.1. Microorganism and Biomass Production

Cyanobacterial biomass was obtained from *S. salina* LEGE 06155, sourced at the Blue Biotechnology and Ecotoxicology Culture Collection (LEGE-CC). *S. salina* biomass production was performed in 5-L glass flat bottom round flasks (4.8 L of working volume), using Z8 culture medium [solution A (10 mL) composed by NaNO_3_ (46.7 g·L^−1^), Ca(NO)_3_.4H_2_O (5.9 g·L^−1^); solution B (10 mL) composed by MgSO_4_.7H_2_O (2.5 g·L^−1^); K_2_HPO_4_ (3.1 g·L^−1^); Na_2_CO_3_ (2.1 g·L^−1^); Fe-EDTA solution (10 mL) composed of FeCl_3_.6H_2_O (2.8 g in 100 mL), HCl (0.1 N)] and EDTA-Na (10 mL) [composed by EDTA (3.9 g in 100 mL) and NaOH (0.1N)] and solution of micronutrients (1 mL) [composed of NaWO_4_.2H_2_O (0.33 g·L^−1^), (NH_4_)6Mo7O_24_.2H_2_O (0.88 g·L^−1^), KBr (1.2 g·L^−1^), KI (0.83 g·L^−1^), ZnSO_4_.7H_2_O (2.87 g·L^−1^), Cd(NO_3_).4H_2_O (1.55 g·L^−1^), Co(NO_3_)_2_.6H_2_O (1.46 g·L^−1^), CuSO_4_.5H_2_O (1.25 g·L^−1^), NiSO_4_(NH_4_)_2_SO_4_.6H_2_O (1.98 g·L^−1^), Cr (NO_3_)_3_.9H_2_O (0.41 g·L^−1^), V_2_O_5_ (0.089 g·L^−1^), Kal(SO4)2.12H2O (9.48 g·L^−1^), H_3_BO_3_ (3.1 g·L^−1^), MnSO4.H_2_O (2.23 g·L^−1^); and vitamin B12 (10 μg·L^−1^ in medium final concentration)] [34] supplemented with 25 g·L^−1^ NaCl, and with pH adjusted to 7.20 ± 0.05. The cultures were kept in batch mode at 25 °C, under a light intensity of 100 µmolphotons·m^−2^·s^−1^ supplied by fluorescent lamps (Biolux, Osram), and a light/ dark cycle of 16 h:8 h (L:D). Continuous agitation was provided by air bubbling at the bottom of the cultures, using an airflow of 0.75 L_air_ ·Lculture^−1^·min^−1^. For each batch, all inocula were established with initial optical density of 0.1 (OD = λ_680_ nm − λ_750_ nm), and cultures were harvested at 22 days. The wet biomass was then freeze-dried and kept in a vacuum desiccator prior to further use.

### 2.2. Experimental Design

To optimize extraction yields and content of chl *a*, total carotenoids, total PBPs—including PC, APC, PE, and antioxidants from ethanolic and aqueous extracts, a factorial design was performed by resorting to the Box–Behnken model. The influence of three factors was analyzed: biomass to solvent ratio (1.5–8.5 mg·mL^−1^), duty cycle (40–100%), and amplitude (40–100%), at equidistant levels (coded as −1, 0, 1)—as presented in Table 1, for a total of 13 runs (run in triplicate). Design-Expert 12 software (Stat-Ease, Minneapolis, MN, USA) was used to construct and analyze design and model results [35].

A second order polynomial (quadratic model) was used to fit the objective functions (quadratic model). A multiple regression of experimental data was performed to acquire the coefficients and equation used to predict the response—besides analyzing the interaction effect between factors and determining the optimum conditions.

The second order polynomial equation that was used is expressed as follows:

Y = α_0_ + β_1_A + β_2_B + β_3_C + γ_1_AB + γ_2_AC + γ_3_BC + ⍵_1_A^2^ + ⍵_2_B^2^ + ⍵_3_C^2^ (1)

in which Y is the predicted response; α_0_ is a constant (intercept); β_1_, β_2_, β_3_, are the linear coefficients; γ_1_, γ_2_, γ_3_, are the interaction coefficients between two factors; and ⍵_1_, ⍵_2_, ⍵_3_ are the quadratic coefficients. A, B and C serve as independent variables, viz. biomass to solvent ratio, duty cycle, and amplitude, respectively.

The fitted polynomial equation was illustrated as surface and contour plots. To ascertain the goodness of fit, the regression coefficient, R^2^, was calculated for every objective function elected. In addition, the best conditions attained for each objective function were scored, in terms of desirability (from 0 to 1)—the higher the desirability, the higher the score.

### 2.3. Extraction Process

#### 2.3.1. Ultrasound Processor and UAE

**Figure 1:**
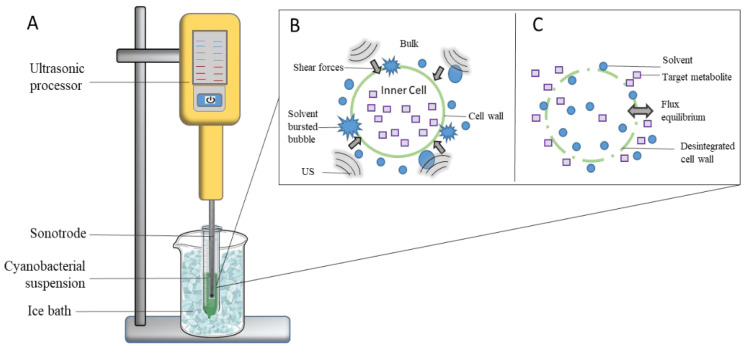
Schematic representation of experimental ultrasound-assisted extraction set-up (**A**). Hypothetical effect of ultrasound (US) via shear forces, and consequent cavitation effect within solvent upon the cyanobacterial cell walls (**B**); and cyanobacterial cell wall disruption and disintegration, concomitant with release of target metabolites (**C**).

To perform the extraction of bioactive pigments and antioxidants compounds, a transportable laboratory ultrasonic processor (Ultrasonic Processor UP200Ht, Hielscher Ultrasonics, Teltow, Germany), with 300 mm × 190 mm × 90 mm, was used—operated at 26 kHz, with rated power of 200 W and equipped with a sonotrode S26 d1 probe. Before each extraction, all samples were hand-homogenized, and then the tip probe was immersed in 2/3 of the total solvent height (4.5 cm). All extractions were performed at room temperature, but samples were placed in ice to avoid overheating (and consequent degradation of pigments). A schematic representation of the experimental setup is shown in Figure 1A. The hypothetical action of US cavitation by solvent within the cyanobacterial cell walls is also depicted in Figure 1B; whereas disruption of the cell wall and release of target metabolites with flux equilibrium are depicted in Figure 1C. For the extraction, 7.5 mg, 25 mg, and 42.5 mg of dried biomass (according to experimental design, see Table 1) was weighed in 15 mL-Falcon tube, and added with 5 mL of solvent.

A first extraction was performed with ethanol—aiming at extracting chl *a* and carotenoids; the samples were centrifuged (2744× *g* for 10 min) and ethanolic extracts collected and stored under darkness at −4 °C; then using the remaining pellet. Another extraction was performed with water, under the same experimental conditions, aimed at extracting total PBPs (PC, APC, PE); the sample obtained was centrifuged 1960× *g* for 10 min, and the extract stored for further determination of yield, PBPs pigment, and antioxidant capacity. This procedure envisaged not only recovery of important bioactive pigments (chl *a* and carotenoids) in terms of antioxidant capacity, but also allowed better access to PBPs in the following aqueous extraction. In a previous work, PBPs content (particularly PC) was shown to increase when biomass was subjected to one or two previous extractions with organic solvents (i.e., ethanol) in *S. salina* [5].

A combination of different duty cycles (expressed as %) were applied, i.e., 40, 70, and 100%, for a total of 2 min as extraction time (see Table 1); the total cycle time comprised a pulse duration and a pulse interval. The amplitude (expressed as %) was also applied in the range 40–100%, (see Table 1). Amplitude percentage refers to the percentage of maximum power used in the equipment.

#### 2.3.2. Classical Extraction of Bioactive Pigments

To validate the use of UAE for bioactive pigment extraction, a comparison was performed with another two extraction methods—classical extraction and bead beater-based homogenization (*Precellys*) extraction. The best results of B/S ratio achieved in optimization for the PC extraction was used for this comparison. Other bioactive pigments, including chl *a*, total carotenoids and APC, PE, total PBPs, extract yields and AOX capacity were also analyzed under a biorefinery perspective. All assays were performed as described in the section of analytical methods.

Biomass was weighted in a 15 mL-Falcon tube (7.5, 25 or 42.5 mg, see Table 1), and 5 mL of ethanol (96% *v*/*v*) was added for chl *a* and total carotenoid extraction. The extraction occurred at 50 °C (in a heating plate), with magnetic stirring over 1 h (with the sample protected from light by aluminum foil). The extract was then centrifuged at 2744× *g* for 10 min, and the supernatant kept under darkness and stored at −4 °C for further analysis. A volume of 5 mL of water was added to the pellet, for successive extraction. The samples were then subjected to 5 cycles of 2 h of freeze/thawing, and another two overnight cycles, until the PC content attained 10% (or less) concentration during the 1st cycle. Aqueous extracts were centrifuged at 1960× *g* for 8 min, and supernatants were kept under dark and stored at −4 °C until further analysis.

The methodology resorting to *Precellys* homogenizer (Bertin Technologies, Montigny-le-Bretonneux, France) was previously described for *S. salina* pigment extraction [5]. Briefly, glass beads and 5 mL ethanol were added to freeze dried biomass; homogenization consisted of a 6 min-cycle at 8000 rpm (30 s homogenization, with 40 s of stopping intervals). Another 5 mL of water and samples were added to the pellet, vortex stirred over 20 s, and finally centrifuged at 1960× *g* for 8 min.

### 2.4. Analytical Methods for Extract Composition Determination

#### 2.4.1. Determination of Chl *a* and Total Carotenoids

Total carotenoids and chl *a* content were spectrophotometrically (Shimadzu UV-1800, USA) determined in ethanolic extracts, according to Lichtenthaler and Buschmann (2001) [36]. Absorption was read at λ_470_, λ_664_, and λ_648_ nm, and content calculated as follows:

Chl *a* (μg·mL^−1^) = (13.36 A_664_) − (5.19 A_648_) (2)

Total de carotenoids (μg·mL^−1^) = (1000 A_470_) − (2.13 Chl *a*)/209 (3)

All samples were analyzed as chemical triplicates. The concentration of these pigments was expressed as milligram per gram of dry weight (mg·g_DW_^−1^).

#### 2.4.2. Determination of Phycobiliproteins

In water extracts, PBP content, namely of phycocyanin (PC), allophycocyanin (APC), and phycoerythrin (PE), was assessed spectrophotometrically by measuring absorbance at λ_562_, λ_615_, and λ_652_ nm, and then applying Bennett and Bogorad’s equations [37], viz.:

(4) $$\mathrm{PC}\;(\mathrm{mg}\cdot {\mathrm{mL}}^{-1})=(A_{615})\;-\;0.474\;A_{652})/5.34$$

(5) $$\mathrm{APC}\;(\mathrm{mg}\cdot {\mathrm{mL}}^{-1})=[(A652)\;-\;(0.208\;A615)]/5.09$$

(6) $$\mathrm{PE}\;(\mathrm{mg}\cdot {\mathrm{mL}}^{-1})=[(A562)\;-\;2.41(\mathrm{PC})\;-0.849\;\left(\mathrm{APC}\right)]/9.62$$

All samples were analyzed as chemical triplicates. The PBP concentrations were expressed as mg·g_DW_^−1^.

### 2.5. Extract Yield Determination

Both ethanol and water extraction yields (γ) were determined as follows:

(7) γ (%DW)=(Mextract/Vsolvent) /(Binital) × 100%

in which M_extract_ corresponds to the weight of mass of extract after solvent evaporation (for ethanolic extract) and lyophilization (for water extract); V_solvent_ stands for the volume of solvent used in the extraction, and B_initial_ for the weight of cyanobacterial biomass initially used in the assays.

### 2.6. Determination of Antioxidant (AOX) Capacity

The total AOX capacity of the ethanolic and successive aqueous extracts was evaluated spectrophotometrically via the ABTS^•+^ assay, in a microplate spectrophotometer reader (Multiscan Go, ThermoFisher, Waltham, MA, USA), according to Guedes et al. (2013) [38], and following adaptation to spectrophotometer plate by Granados-Guzman et al. (2017) [39]. ABTS^•+^ is a radical-scavenging method valid for detection of both lipophilic and hydrophilic antioxidant compounds [40]. Briefly, ABTS radical cation was produced by reacting potassium persulfate (SigmaAldrish, St. Louis, MO, USA) (0.66 mg·mL^−1^) and ABTS (SigmaAldrish, St. Louis, MO, USA) (3.84 mg·mL^−1^). For the assay, 63 µL of extract sample was added to 180 µL of ABTS solution, so that the final absorption lay between 0.68 and 0.72. All samples were incubated for 6 min, before absorbance was read, in triplicate, at λ_734_ nm.

For all assays, the percent inhibition was as follows:

% inhibition = [(Abs_sample_ − Abs_blanck sample_) − Abs_control_]/Abs control × 100% (8)

where Abs_sample_ is extract absorbance, Abs_blanck_ is solvent absorbance of ABTS reagent, and Abs_control_ is absorbance of ethanol or water. Results were plotted as two calibration curves, previously established with Trolox dissolved in ethanol and water, respectively. The antioxidant capacity was expressed in mg Trolox equivalents (TE) per gram of dry weight (mg_TE_·g_DW_^−1^).

### 2.7. Statistical Analysis

Statistical analysis was performed using Design-Expert 12 software [35], and based on fit of a quadratic polynomial model—containing linear, quadratic, and interaction coefficients, to the experimental data. Analysis of variance (ANOVA) was used to assess the statistical significance of the fit. ANOVA (at 95% confidence level) was carried out to ascertain significance of model terms. The data were subjected to regression analysis, using least squares methodology, to generate the best equation that provided the response values as a function of the independent variables.

## 3. Results

### 3.1. Design Experiment

A total of 13 experimental runs were performed according to Box–Behnken design, in order to find the optimal conditions in terms of B/S ratio, duty cycle, and amplitude—corresponding to the highest content of several bioactive pigments of interest, in particular PC. For all parameters analyzed—pigment content, extraction yields, and AOX capacity, the model chosen was statistically significant with a *p*-value < 0.05 (Table 2). The regression model exhibited a good fitness, particularly for PC, APC, PE, total PBPs, yield of water extraction, and antioxidant capacity in ethanolic extract—with R^2^ ranging from 0.70 to 0.96; this unfolded a reasonably high degree of correlation, between experimental and predicted values (Table 2).

### 3.2. Bioactive Pigment Extraction Yields

#### 3.2.1. Chlorophyll *a* Extraction Yields

The extraction yields of chl *a* were determined for each experimental condition as per the factorial design. The experimental values of chl *a* ranged between 1.07 ± 0.05 and 4.46 ± 0.21 mg·g_DW_^−1^ (Table 3).

**Figure 2:**
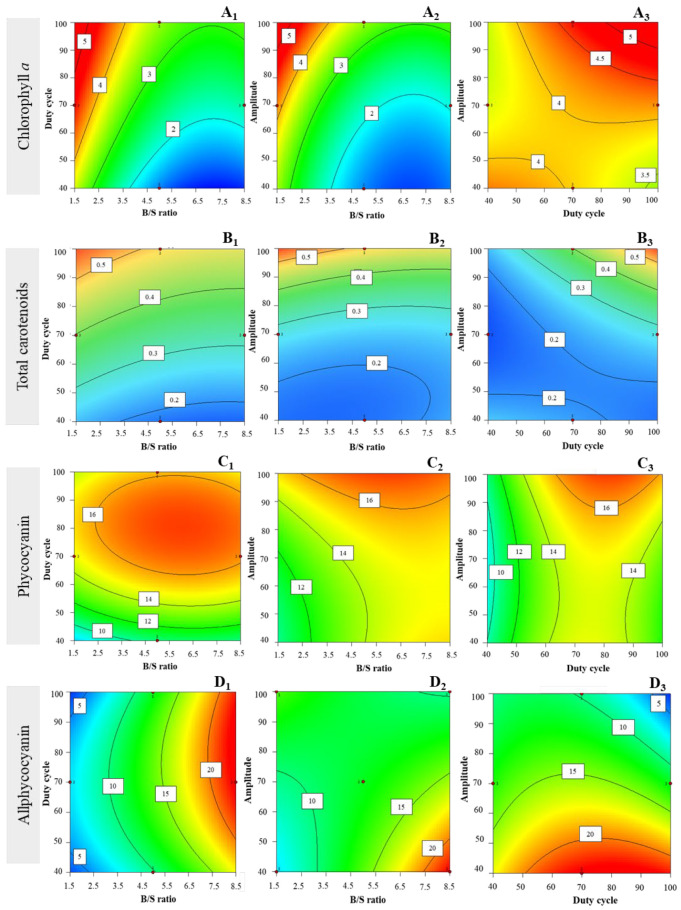

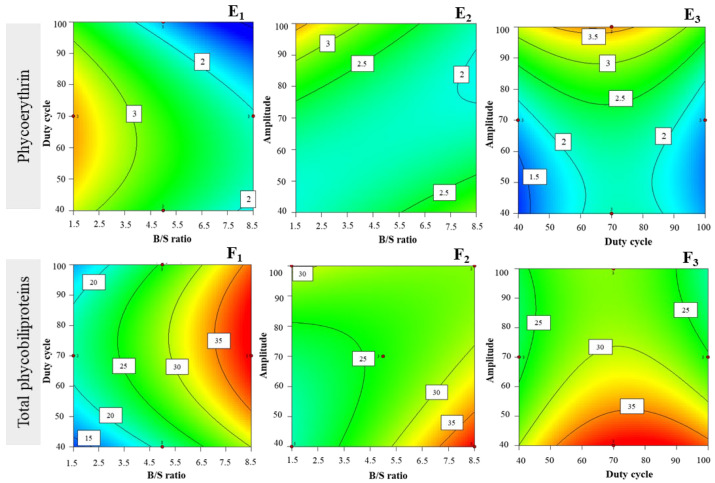
Response surface plots for bioactive pigments (mg.g_DW_^−1^), with corresponding interaction effect between parameters B/S ratio, duty cycle, and amplitude—for chl *a* after setting (**A_1_**) amplitude = 100%, (**A_2_**) duty cycle = 100%, and (**A_3_**) B/S ratio = 1.5 mg·mL^−1^; for total carotenoids, after setting (**B_1_**) amplitude = 100%, (**B_2_**) duty cycle = 100%, and (**B_3_**) B/S ratio = 1.5 mg·mL^−1^; for phycocyanin (PC), after setting (**C_1_**) amplitude = 100%, (**C_2_**) duty cycle = 80%, and (**C_3_**) B/S ratio = 6 mg·mL^−1^; for allophycocyanin (APC)**,** after setting (**D_1_**) amplitude = 40%, (**D_2_**) duty cycle = 70%, and (**D_3_**) B/S ratio = 8.5 mg·mL^−1^; phycoerythrin (PE), after setting (**E_1_**) amplitude = 100%, (**E_2_**) duty cycle = 60%, and (**E_3_**) B/S ratio = 1.5 mg·mL^−1^; and total of phycobiliproteins (PBPs), after setting (**F_1_**) amplitude = 40%, (**F_2_**) duty cycle = 70%, and (**F_3_**) B/S ratio = 8.5 mg·mL^−1^.

For chl *a*, the response surface plots showed that setting high amplitudes (i.e., 100), the interaction effect between high duty cycle (i.e., 100%) and low B/S ratio (i.e., 1.5 mg·mL^−1^) increases chl *a* yields (Figure 2A_1_). The increase in amplitude with low B/S ratio positively influenced the yield of chl *a* (Figure 2A_2_); hence, by setting low B/S (i.e., 1.5 mg·mL^−1^), the effect of increased amplitude and increased duty cycle positively impacted extraction of chl *a* (Figure 2A_3_). The B/S ratio per se also influenced significantly (*p* < 0.05) the extraction yields of chl *a* (data not shown).

#### 3.2.2. Total Carotenoids Extraction Yields

The total carotenoid content ranged from 0.16 ± 0.00 to 0.57 ± 0.06 mg·g_DW_^−1^ (see Table 3). By setting high amplitude (i.e., 100%), it was observed that a low B/S ratio (i.e., 1.5 mg·mL^−1^) and a high duty cycle (i.e., 100%) had a positive impact upon total carotenoid yield, with a non-significant first-order interaction (*p* > 0.05). The model also confirmed interaction effects between high amplitude (i.e., 100%) and low B/S ratio (i.e., 1.5) (Figure 2B_1_); in addition, surface plots showed that the interaction effect between amplitude and duty cycle was higher when setting a lower B/S ratio (i.e., 1.5) (Figure 2B_2_)—as it positively influenced total carotenoid yields (Figure 2B_3_).

#### 3.2.3. Phycocyanin Extraction Yields

PC yields obtained ranged between 6.26 ± 0.27 and 17.22 ± 0.54 mg·g_DW_^−1^ (Table 3). The response surface plots indicated that under a high amplitude, the interaction effect between intermediate duty cycles (i.e., 80%) and B/S ratio (i.e., 6 mg·mL^−1^) positively impacted extraction yields (Figure 2C_1_). In addition, at higher amplitude and intermediate B/S ratio, there was a tendency for increase of PC extraction yields when the duty cycles were set at intermediate levels (i.e., 80%) (Figure 2C_2_). The interaction of higher amplitudes with intermediate duty cycles seemed to produce increased yields of extraction (Figure 2C_3_), after setting an intermediate B/S ratio (i.e., 6 mg·mL^−1^). Each factor per se influenced significantly (*p* < 0.05) PC extraction yields (data not shown).

#### 3.2.4. Allophycocyanin Extraction Yields

APC extraction yields ranged between 2.31 ± 0.01 and 21.23 ± 0.88 mg·g_DW_^−1^ (Table 3). The effect of intermediate duty cycles with higher B/S ratios, when set at low amplitude (i.e., 40%), seems to enhance extraction yields of APC (Figure 2D_1_); on the other hand, low amplitude and higher B/S ratios positively impacted extraction yield (Figure 2D_2_)—and an interaction effect was observed with the duty cycle, under several ranges (in particular intermediate levels, i.e., 70–80%), when B/S ratio was set at higher level (i.e., 8.5 mg·mL^−1^) (Figure 2D_3_).

#### 3.2.5. Phycoerythrin Extraction Yields

PE extraction yields ranged between 1.37 ± 0.15 and 3.98 ± 0.07 mg·g_DW_^−1^ (Table 3). The interaction effects between variables were not so well defined, but they seemed to follow a trend when setting higher amplitudes (i.e., 100%), lower B/S ratio (i.e., 1.5 mg·mL^−1^), and intermediate levels of duty cycle (i.e., 60–70%)—which tend to increase extraction yields of PE (Figure 2E_1_). In the same way, after setting duty cycle to intermediate levels, a trend was found for low B/S ratio toward increase of extraction yields (Figure 2E_2_), as well as for intermediate levels of duty cycle and higher amplitude (Figure 2E_3_).

#### 3.2.6. Total Phycobiliprotein Extraction Yields

Total PBP extraction yields reached between 13.75 ± 1.73 and 36.90 ± 1.12 mg·g_DW_^−1^ (Table 3). After setting low amplitude (i.e., 40%), the interaction between high B/S ratio and intermediate levels of duty cycle apparently enhances extraction yields (Figure 2F_1_). The effect of interaction between amplitude and B/S ratio is not so relevant, yet a trend toward higher extraction yields of PBPs is observed at higher B/S ratio (i.e., 8.5 mg·mL^−1^) and lower amplitude (i.e., 40%) (Figure 2F_2_). On the other hand, interaction between amplitude and duty cycle at low amplitudes (i.e., 40), with intermediate duty cycles (70–80%) favors high extraction yields (Figure 2F_3_).

### 3.3. Extract Yields

#### 3.3.1. Ethanolic Extracts Yield

**Figure 3:**
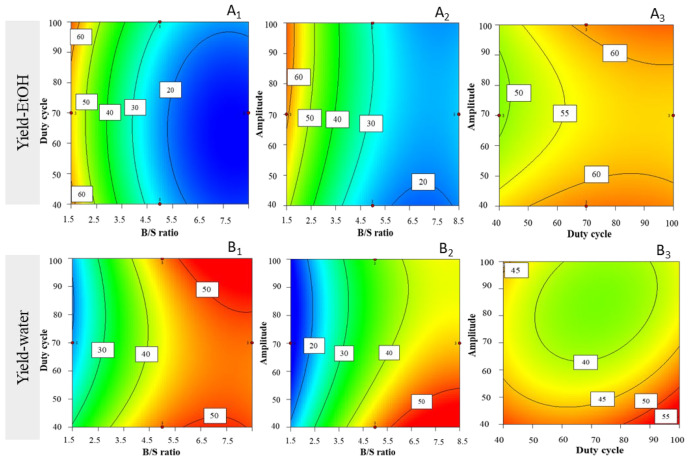
Response surface plots for yields of extracts (%_DW_), with corresponding interaction effect between parameters B/S ratio, duty cycle, and amplitude—for ethanolic (EtOH) extract after setting (**A_1_**) amplitude = 100%, (**A_2_**) duty cycle = 100%, and (**A_3_**) B/S ratio = 1.5 mg·mL^−1^; and for water extracts, after setting (**B_1_**) amplitude = 40, (**B_2_**) duty cycle = 100, and (**B_3_**) B/S ratio = 8.3 mg·mL^−1^.

The general yield of extracts produced was also assessed via the quadratic model, with experimental values ranging within 14.92 ± 1.35 and 69.71 ± 0.00%_DW_ (mass dry weight) (Table 3). The surface plot areas did not unfold significant interactions between tested variables, yet some trends regarding EtOH yield were found. Higher duty cycles (i.e., 100%), concomitant with low B/S ratios (i.e., 1.5 mg·mL^−1^) (Figure 3A_1_) show a tendency for increasing EtOH yields; a similar tendency was observed for higher amplitude (i.e., 100%) and lower B/S ratio (i.e., 1.5 mg·mL^−1^) (Figure 3A_2_), or after setting a low B/S ratio. The trend of enhanced EtOH yields is graphically shown for both high amplitude and high duty cycles (i.e., 100%) (Figure 3A_3_).

#### 3.3.2. Water Extracts Yield

The general yield of extracts produced for successive aqueous extracts was also assessed, and experimental values ranged between 14.68 ± 0.31 and 51.37 ± 0.72%_DW_ (Table 3). The surface plot graphs unfolded an interaction between high duty cycles with higher B/S ratio (i.e., 8.3 mg·mL^−1^), after setting low amplitude (i.e., 40%), thus resulting in higher water yields (Figure 3B_1_). When setting high duty cycles (i.e., 100%), higher B/S ratios (i.e., 8 mg·mL^−1^) combined with lower amplitude (i.e., 40%) positively influenced aqueous extraction yield (Figure 3B_2_). On the other hand, the influence of lower amplitude and higher duty cycles (when setting B/S ratio to 8.3 mg·mL^−1^) enhanced extraction yields (Figure 3B_3_). A similar trend was observed for lower duty cycle, yet not so pronounced.

### 3.4. Antioxidant Capacity (AOX)

#### 3.4.1. Antioxidant Capacity of Ethanolic Extract Yield

**Figure 4:**
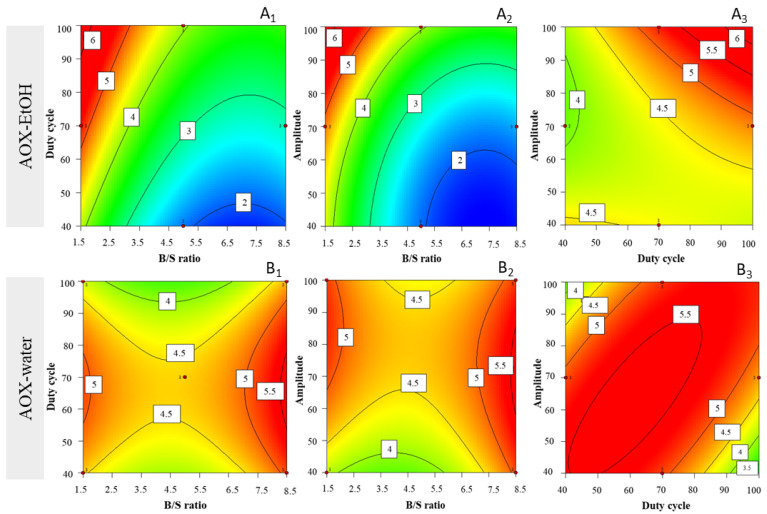
Response surface plots for antioxidant capacity (AOX) of extracts (mgTE·mgDW), with corresponding interaction effect between parameters B/S ratio, duty cycle, and amplitude—for AOX of ethanolic (EtOH) extract, after setting (**A_1_**) amplitude = 100%, (**A_2_**) duty cycle = 100%, and (**A_3_**) B/S ratio = 1.5; and for AOX of water extracts, after setting (**B_1_**) amplitude = 70%, (**B_2_**) duty cycle = 70%, and (**B_3_**) B/S ratio = 8.2 mg·mL^−1^.

Antioxidant capacity in ethanolic extracts was indirectly measured as Trolox equivalents (ABTS method) and considered as objective function with a similar model. The experimental values ranged between 1.72 ± 0.02 and 5.27 ± 0.10 mg_TE_·g_DW_^−1^ (Table 3). The model indicated that the best conditions to obtain ethanolic antioxidant compounds are B/S ratio = 1.5 mg·mL^−1^, duty cycle = 100%, and amplitude = 100%. In the surface plots associated to high amplitude (i.e., 100%), for lower B/S ratios, and higher duty cycles, a trend is visible of increasing antioxidant capacity (Figure 4A_1_); a similar interaction was observed for low B/S ratio (i.e., 1.5 mg·mL^−1^) and high amplitude (i.e., 100%), yet the interaction seems more notorious for these two parameters (Figure 4A_2_). By establishing a low B/S ratio (i.e., 1.5 mg·mL^−1^), the interaction effect regarding higher amplitude (i.e., 100%) and higher duty cycle (i.e., 100%) seems to impact positively upon antioxidant capacity in ethanolic extracts (Figure 4A_3_).

#### 3.4.2. Antioxidant Capacity of Water Extract Yield

Antioxidant capacity in aqueous extracts was also indirectly expressed as Trolox equivalents and introduced as well as objective function to the quadratic model under scrutiny. The experimental values ranged between 1.54 ± 0.04 and 5.28 ± 0.07 mg_TE_·g_DW_^−1^ (Table 3). The conditions that maximize extraction yields in terms of antioxidant aqueous compounds are B/S ratio = 8.2 mg·mL^−1^, duty cycle = 70%, and amplitude = 70%. When setting intermediate amplitudes (i.e., 70%), the surface plot suggests that AOX compounds are favored by high B/S ratio (i.e., 8.2) and intermediate duty cycles (i.e., 70%) (Figure 4B_1_). On the other hand, when setting intermediate duty cycle (i.e., 70%), for higher B/S ratios and intermediate amplitudes, the yields of AOX compounds increased (Figure 4B_2_). Once again, after establishing higher B/S ratio (i.e., 8.2 mg·mL^−1^), the interaction between amplitude and duty cycle was quite significant—and AOX compound extraction yields were favored by intermediate levels (i.e., 70%) of either factor (Figure 4B_3_).

### 3.5. Model Optimum Conditions

For each parameter evaluated, the optimum conditions needed to achieve maximum extraction yields were sought (Table 4). The optimum extraction conditions for PC in the water extracts were B/S ratio of 6, duty cycle of 80%, and amplitude of 100%—leading to a maximum extraction yield of 17.21 ± 0.86 mg·g_DW_^−1^ (desirability of 0.942).

The best conditions to achieve high APC extraction yields were similar to those associated with total PBPs. A B/S ratio of 8.5 mg·mL^−1^, combined with a duty cycle of 70% and an amplitude of 40% (desirability = 1), led to maxima of 22.82 ± 2.52 mg·g_DW_^−1^ and 38.97 ± 2.74 mg·g_DW_^−1^, respectively. Conversely, the best conditions to improve PE yields were B/S ratio of 1.5 mg·mL^−1^, duty cycle of 60%, and amplitude of 100%—with estimated maximum of 3.61 ± 0.32 mg·g_DW_^−1^. In terms of general yields of aqueous extract, the model predicted a maximum of 54.96 ± 2.72%_DW_, at B/S ratio of 8.3 mg·mL^−1^, duty cycle of 100%, and amplitude of 40%. With regard to AOX compounds in water extracts, the maximum estimated by the model was 5.44 ± 0.05 mg_TE_·g_DW_^−1^, with B/S ratio of 8.2 mg·mL^−1^, duty cycle of 70%, and amplitude of 70%.

Regarding ethanolic extracts, encompassing chl *a*, total carotenoids, and AOX compounds, the best conditions to obtain higher yields were similar—with B/S ratio of 1.5 mg·mL^−1^, duty of 100%, and amplitude of 100%. The best conditions, and corresponding maxima are presented in Table 3.

### 3.6. Comparison of Ultrasound-Assisted Extraction of Phycocyanin and Conventional Extraction

For comparison with UAE, freeze/thawing (classical) and *Precellys*-based extractions were performed. The best conditions found in terms of B/S ratio were accordingly chosen for extraction of PC (i.e., B/S ratio = 6 mg·mL^−1^, which means 30 mg of biomass for 5 mL of solvent). The high content of PC in *S. salina* [5], and the high commercial interest of this pigment justify those selected conditions; chl *a*, total carotenoids, total PBPs, APC, PE, general yields, and antioxidant capacity were also determined, in order to understand the yields for these parameters within a biorefinary perspective (Table 5).

For PC extraction yields, especially when comparing with classical extraction, the maximum predicted by the model was slightly higher (more 12.5%), although such differences were not significant (*p* > 0.05); when compared to *Precellys*-based process, a 47.8%-increase was observed.

Regarding aqueous extracts, the highest extraction yields in terms of APC, PE and total PBPs were attained with freeze/thawing classical extraction. On the other hand, the yields of aqueous extract and corresponding AOX capacity were higher with UAE than the other methods, but in most cases showed no significant differences (*p* > 0.05). For ethanolic extracts, the general yields of extract and of chl *a,* were significantly higher than UAE (*p* < 0.05). Regarding total carotenoids and AOX of ethanolic extract, the optimum yields were enhanced with UAE, but with no significant differences relative to the classical method (*p* > 0.05).

## 4. Discussion

A UAE has been reported as efficient separation method, characterized by high rates of success in extraction of several biomolecules, from various microalgal sources, e.g., lutein [41] and chl *a* [22] from *Chlorella vulgaris*; carotenoids and lipids from *Heterochlorella luteoviridis* [20]; β-carotene and proteins from *Arthrospira platensis* [21]; phenolic compounds from *Tetraselmis* sp. [28]; PBPs from *Oscillatoria* sp. [42]; and PC from *Cyanidium caldarium*, and PE from *Porphyridium* spp. [43]. However, the efficiency of this method is highly dependent on the type of microalga matrix (with cell wall robustness and porosity playing a role), target metabolite, or solvent employed. The selection of solvents plays a crucial role in terms of extraction efficiency; they are supposed to assure sufficient solubility of metabolites of interest [44]. Ethanol and water were selected for this study, because they are both GRAS solvents, which means that they have low toxicity, and can thus be widely applied in food and nutraceutical industries, further to their low cost. In addition, successive extractions with an organic solvent meant to extract more lipophilic components (e.g., chl *a* and carotenoids) right after extraction with a more polar solvent (e.g., phosphate buffer saline of water), can favor extraction of PBPs, particularly PC [5]. Since UAE may exhibit low selectivity [2,45], the same strategy was applied in this study with a two-phase extraction; the first stage of ethanolic extraction may be viewed as biomass pre-treatment—and has indeed been reported to positively impact the successive extraction efficiency [44].

### 4.1. Effect of Different Factors in Bioactive Pigments, Yields and AOX

B/S ratio, duty cycle, and amplitude during UAE had different impacts upon extraction yields of bioactive pigments, yields, and AOX capacity. Overall, chl *a*, total carotenoids, general yield, and AOX-EtOH yields for ethanolic extracts were influenced by consistently lower B/S ratios (i.e., 1.5 mg·mL^−1^) than those in the water extracts (except for the PE), with similar duty cycle and amplitudes. In terms of B/S ratios, use of less biomass (in this case, 7.5 mg for 5 mL of solvent) may allow more efficient penetration of solvent into the cyanobacterial matrix, and thus a higher solubilization of the target compounds—as an outcome of the bubble cavitation process induced by ultrasound. Use of more biomass, even after previous vigorous homogenization of the sample, can at some point lead to a small deposit of biomass on the bottom of the container and thus hamper uniform and total penetration of the matrix by solvent during extraction. The same was not observed in the successive extractions with water, because the cyanobacterial cell walls were probably weakened as the biomass had already been pre-soaked in organic solvent. For higher B/S ratios, permeation by the aqueous solvent is facilitated, and target compounds (e.g., PC, PBPs) are more easily exposed. Literature reports are controversial with regard to how B/S ratio influences extraction. For instance, a study to optimize extraction of lutein from *Chlorella vulgaris* tested different solvent-to-solid ratios, say 10, 30, and 50 mL·g^−1^; and 31 mL·g^−1^ appeared as best ratio to improve lutein recovery (EtOH 90% (*v*/*v*), at 37.7 °C and 162 min of extraction time—with an extraction yield of 3.16 ± 0.03 mg·g^−1^ wet biomass [41]. Another study reported that PBP from *Oscillatoria* sp., extracted with Britton–Robinson buffer (0.05 M) and subjected to B/S ratios of 0.15, 0.2, and 0.25 mg mL^−1^, was maximum at a B/S ratio of 0.2 mg·mL^−1^ [42].

In terms of ethanolic extracts, the highest values for both amplitude and duty cycle (i.e., 100%) were found associated to the maximum yields of extraction of chl *a*, total carotenoids, yield, and AOX. This trend, in terms of duty cycle, can be seen as a limitation in extraction time—at least for the first stage of extraction. The maximum duty cycle showed a clear trend of increasing yields of pigments and general yield when B/S ratio was low, and a similar result was perceived for amplitude. The latter is related to maximum percentage input of power/energy in the system, meaning that the higher the amplitude, the higher the energy transmitted to the solvent, and thus the higher the energy used to disrupt cyanobacterial cells [29].

For aqueous extracts, and focusing on PC extraction yields, the model showed an intermediate B/S ratio (i.e., 6 mg·mL^−1^)—with duty cycle of 80% and highest amplitude of 100% giving the best results. The interaction effect between intermediate B/S ratios and duty cycles was of particular significance, because duty cycles under 70% and above 90% did not influence extraction yields of PC in a positive manner. The fact that biomass was already pre-soaked in ethanol as per the preceding extraction can contribute to increase the B/S ratio; the intermediate duty cycle toward optimization of extraction yields of PC is possibly related to degradation of this pigment at higher duty cycles (i.e., the more effective time of extraction). It is known that a longer sonication time increases initially, but then reduces extraction efficiency [46]. The duty cycles can help to make some time intervals during the extraction process—helpful to avoid overheating of the sample, and consequently prevent degradation of more thermolabile bioactive compounds [2,46,47]. Temperature is a relevant parameter upon amount of PC extracted after disintegration by ultrasound [32,47]. Fratelli et al. (2021) noted that heat development can occur, and might constitute a drawback when applying UAE to PC extraction [32]—chiefly because heat-related protein denaturation is responsible for activity loss in PC. A higher temperature helps in desorbing compounds present in the matrices (e.g., PC), and increases their solubility in the solvent; in addition, it reduces solvent viscosity, thus increasing diffusivity of target metabolite in the solvent. However, when temperature goes beyond a maximum threshold, cavitation becomes less effective [46]. This can cause thermal vibration and affect the native or functional structure of PC. Note that its structure is held together by an intricate balance of covalent (ion dipole and hydrogen bonds) and non-covalent (hydrophobic and van der Waals) interactions, which lead to non-proteolytic modification—and thus give rise to changes in chemical, physical, and biological properties [24,48]. Some studies have indeed confirmed that periods of cooling during extraction enhance PC extraction yields [32,48]; which in this study can be promoted by intermediate duty cycles. Other studies have employed minimal time of extraction, not exceeding a few minutes (e.g., 3 min) to minimize thermal destabilization of PC [49].

Regarding the results of other PBPs (APC, PE), the optimal conditions predicted were significantly different—as well as the interaction effects between factors. A major observation is that the best conditions to maximize APC (and total PBPs) extraction yields were similar; high B/S ratio combined with intermediate duty cycle (i.e., 70%) and low amplitude (i.e., 40%) favor them. Following the previous discussion, the stability of APC may get compromised owing to extended duty cycles, combined with high amplitudes or energy cycles; in fact, there is a chance for overheating the sample, which may lead to degradation of those pigments. As outlined above, PBPs are sensitive to temperature [50]. Stability of both PC and APC has been recorded up to 40–50 °C [24]; there is an increasing and sudden degradation rate when those pigments are subjected to 60 to 80 °C [51,52]. Although the temperatures of samples were not measured, samples became greyish, instead of being blue, and also formed foam (data not shown) under more extreme conditions (e.g., when testing DT = 100% and A = 100%). Hence, it can be hypothesized that overheated samples entertain PBP denaturation—which influences directly extraction yields and interaction effects. On the other hand, some trends were found—in particular low B/S ratio (i.e., 1.5 mg·mL^−1^, lower than the other PBPs), combined with intermediate duty cycle (i.e., 60%) and high amplitude (i.e., 100%), as favorable to enhance PE extraction yields. The same logic applies to PE best extraction yields; despite the amplitude being higher (which could effectively lead to higher extraction efficiency, as more energy is displaced in the sample), an intermediate duty cycle cut the effective operation time to almost half; this may suffice to not overheat the sample and can be more effective in preserving PE stability throughout extraction. Unlike the other extraction conditions of PBPs, the B/S ratio was lower; this realization may relate to biomass being more available for extraction.

Enhanced general yields for aqueous extracts, as well aqueous AOX compounds were favored by high B/S ratios (i.e., 8.2 and 8.3, respectively). In the first case, the duty cycle was 100% for general yields—yet low amplitude was found best toward improved extraction. This combination may reflect also a pre-treatment performed with ethanol. It could be expected that maximum duty cycle and amplitude would lead to maximum yields, but the fact that pre-treatment “cleans” part of the biomass makes extractable components more available; and the fact that the duty cycle is complete, but the energy applied is lower may contribute to prevent components from degrading by heat. On the other hand, higher B/S ratios, combined with mild conditions of duty cycles and amplitude (both 70%) potentiate higher yields of extraction of AOX compounds. Some of these compounds can be more volatile under more extreme conditions, so mild conditions seem to favor extraction yields thereof.

### 4.2. Comparison of Ultrasound-Assisted Extraction of Phycocyanin to Conventional Extractions

Conventional methods for PC extraction, specifically freeze/thawing and homogenization by bead beater, were compared with values obtained using UAE. Those methods were chosen because they are well established methodologies for PBP extraction and possess high efficiency in cyanobacterium cell disruption and PBP release [2,52,53]. Regarding PC extraction yield, an increment of 12.5% (17.21 ± 0.86 mg·g_DW_^−1^) using UAE was found, under best operating conditions when compared to freeze/thawing (15.30 ± 1.51 mg·g_DW_^−1^), and an increase of 47.8% (11.64 ± 0.08 mg g_DW_^−1^) when compared to *Precellys*-based extraction. Information in the literature is not fully consistent regarding freeze-thawing and UAE—with reports of higher yields of PC for one or another [32,49,54,55]. For instance, Tavanandi et al. (2018) [49] reported PC extraction with classical extraction freeze-thawing (nine cycles) of 119 mg·g_DW_^−1^ for *Arthrospira platensis* dried biomass. They compared this value with UAE and also homogenization, both performed by pre-soaking biomass over 120 min—and found values of 50.1 mg g_DW_^−1^ and 52.26 mg·g_DW_^−1^, respectively. Those authors decided to use UAE as a pre-treatment with other conventional methodologies (e.g., freeze-thawing, maceration), thus positively impacting upon PC extraction. Ores et al. 2016 [54] have compared UAE and freeze/thawing (four cycles) extraction methods for *Arthrospira platensis*, and found no statistical differences between them. However, the value of PC concentration was higher than in *S. salina*; they obtained 90 ± 0.1 mg·g_DW_^−1^ for UAE, and 101 ± 0.2 mg·g_DW_^−1^ for freeze/thawing. Nevertheless, any such comparisons need to take into account the type of microalga/cyanobacterium at stake (for distinct morphology, physiology, metabolites of interest), and the conditions under which biomass is produced (e.g., light, nutrients, temperature, pH) [32].

It is worth mention that the antioxidant capacity of the extracts changed depending on the method applied. For instance, the AOX capacity, for both ethanolic and aqueous extracts, regarding homogenization-based extraction was lower than UAE or classical techniques. Despite general yields being high, extraction of bioactive pigments (i.e., PBPs, carotenoids) was somewhat less efficient, possibly due to some level of degradation during shear-homogenization. On the other hand, low extraction yields were found for ethanolic UAE, yet AOX capacity was highest compared to other methods—a possible outcome of the highest content of total carotenoids extracted by this method.

UAE still presents several advantages over classical techniques. The latter are usually time-consuming, have high energetic demands, and use high amounts of solvent, which make them impractical at large scale, both for economic and environmental reasons [56]. In this study with UAE, PC extraction can be enhanced by spending only 4 min (counting on successive extractions) instead of several hours of freeze-thawing. Similar results were reported by Hadiyanto et al. (2016), who reduced extraction time of PC in *Arthrospira platensis* dry biomass from 10 h (freeze-thawing) to 2 min (UAE) [24]. On the other hand, the volume of solvent involved in UAE process was reduced, thus minimizing generation of additional waste [49,57]; freeze-thawing required five times more volume required by UAE. Furthermore, pulsed energy modes (duty cycles) help achieve a more sustainable process (besides promoting heat dissipation). Pan et al. (2011) found no correlation between yield efficiency and duration of cycle, yet they noted a 50% reduction in electricity consumption in pulse modes [58].

It is worth mentioning that UAE is not very selective [2,19,45]. For instance, Li et al. (2020) reported a problem of contamination of chl *a* in PC aqueous extracts obtained by UAE [45]. Hence, use of a US two-phase extraction, in particular with GRAS solvents, allows a higher selection selectivity of this technique to obtain PC.

**Figure 5:**
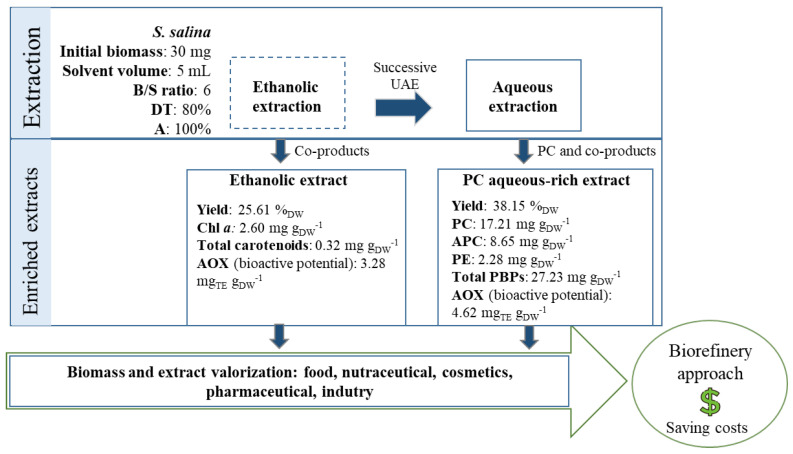
Proposed UAE algal bioprocess for PC extraction towards a biorefinery approach.

Selecting the best conditions to extract PC (B/S ratio = 6 mg·mL^−1^, DT = 80%, and amplitude = 100%), *S. salina* biomass can be valorized for other co-products as proposed in Figure 5. The bioprocess has the advantage of simultaneously obtaining pigments and AOX fractions, by fractionating the lipophilic compounds and aqueous compounds from *S. salina*, thus envisaging a biorefinery approach.

In a first step, the biomass would be subjected to UAE combined with ethanol (a more lipophilic solvent), in order to make PC and other PBPs more available to the next step of extraction. This extraction would allow an ethanolic extract be obtained with bioactive potential (general yield of 25.61%_DW_), and other co-products such as chl *a*, total carotenoids, and AOX compounds with commercial interest. Both chl-related molecules and carotenoids have witnessed an increasing in the market of natural products; for instance, the market of carotenoids used in nutraceutical, cosmetics, food, and feed industries surpassed USD 1.5 billion from 2016–2019, and is expected to reach USD 2 billion (USD) in 2026 [59]. The market of chl is also undergoing expansion; in 2018, it added to around USD 279.5 million, but is expected to attain USD 463.7 million by 2025. Additionally, the extracts possess bioactivity that can be related not only to the presence of carotenoids and chl *a*, but also to other value-added compounds such as phenolic compounds, as demonstrated in a previous study [5]. Those are secondary metabolites with a wide range of chemical structures and form an important group of bioactive components with radical scavenger properties, able to prevent and fight ROS, and bearing antioxidant capacity; hence being attractive for the pharmaceutical and nutraceutical industries.

In a second step of extraction, UAE would be employed in conjugation with water for the successive extraction of PC. It would allow production of water rich-extracts, containing bioactive ingredients—not only PC, but also APC, PE with demand by various industries (e.g., colorant in food, nutraceutical, feed, cosmetic) [2,3]. Aqueous extracts also revealed antioxidant properties, slightly higher than the ethanolic extracts, possibly due to the presence and high content in PBPs, particularly PC and APC, as these compounds are reported to possess antioxidant activity [8]. Phenolic compounds were previously found in *S. salina* successive aqueous extracts—and probably produce a synergetic effect in terms of antioxidant capacity; extracts with these characteristics are quite appealing for cosmetic and nutraceutical industries.

In a broader context, the majority of cyanobacterial bioprocess aim at a single product recovery, and thus fail to meet the major purpose of valorizing biomass as a whole; this leads to non-feasible process and economic failure, owing to the elevated costs of production (i.e., photobioreactor maintenance, low biomass yields,) and downstream processing (i.e., extraction and purification of the target compound) [60]. Thus, increased economic feasibility of a cyanobacterial biorefinery could be achieved, by ultimately coupling the co-production of other compounds with low value, such as lipids for production of biodiesel. The spent biomass can also be processed through thermal conversion and transformed to biofertilizer, or generate other forms of energy such as biomethane, bioethanol, or biohydrogen through fermentation process [61]. This co-generation of biofuel and bioenergy serves as a route to exploit the benefits of production of heat and electricity able to satisfy the energy requirements of production operation and downstream processing units.

Furthermore, successive extraction procedures could be a strategy for implementation based on high to low market value, envisaging a circular bioeconomy that could eventually lead to a minimal-waste biorefinery. Extractions could be performed in successive order to obtain compounds with different chemical properties, by playing with different polarities of the solvents used. Furthermore, the specificity of the solvent may allow a higher purification level of the compound(s) involved. The use of GRAS solvents is of major importance, for making the process greener and more sustainable for foodstuff purposes [2]. The extraction of more than one product from the same biomass may contribute to reduce costs of the process, with minimal energy use.

### 4.3. Box–Behnken Design as Optimization Tool

The Box–Behnken method is a statistical tool for factorial optimization that can help identify each single or interaction effect of operating parameters upon extraction efficiency. This model has proven a reliable strategy to follow, in attempts to optimize different parameters; and has been included in several studies related to cyanobacterial and biomass extraction optimization [41,62,63,64]. For instance, Hilali et al. (2022) have shown the efficacy of this model (as well as an artificial neural network) to improve UAE conditions for PC extraction in *Arthrospira platensis*—using temperature, extraction time, and water addition as parameters, by employing natural deep eutectic solvents. Such type of statistical methodologies can maximize extraction efficiency based on a limited number of experimental runs—unlike traditional studies that focus on one-factor-at-a-time, and thus take longer to implement. Furthermore, the latter cannot detect interactive effects, either synergistic or antagonistic, across the factors studied, without sacrificing reliability [58].

To the best of our knowledge, this is the first study that provides information on the interaction effects of B/S ratio, duty cycle, and amplitude; and accordingly proposes optimum values thereof for UAE of PC from *S. salina*, and other bioactive pigments and antioxidants with commercial interest. The Box–Behnken design seems a simple and fast tool, able to provide informative data suitable for decision-making at large scale processes [55]. Nevertheless, more fundamental studies are still needed to complement this information—since other factors may influence UAE efficiency. In our study, B/S ratio, duty cycle, and amplitude are relevant toward enhancement of PC yields; however, temperature, extraction time, power or frequency should also be accounted for in future studies. Definition of the best extraction conditions of single- or multi-compound recovery will permit enrichment of the portfolio of processes under a biorefinery perspective. However, the full potential of this technology still requires a multidisciplinary effort of biologists, chemists and engineers in attempts to reach large scale, and a more sustainable and economic microalgal bioprocess.

## 5. Conclusions

Optimum operational UAE conditions for PC extraction revealed to be different from those for the other variables here explored (chl *a*, carotenoids, APC, PE, total PBPs, extract yields, and AOX capacity); yet UAE successive extraction with GRAS solvents, ethanol and water, in those conditions can help upgrade the cyanobacterial extracts for other bioactive compounds of interest to the market of natural products (e.g., carotenoids). This could be an interesting strategy to be implemented—for supporting reutilization of biomass, with minimal waste, ultimately prone to a more sustainable and economically feasible process focusing on the biorefinery and circular bioeconomy concepts.

## Figures and Tables

**Table 1:** Experimental factorial design followed, with processing conditions chosen for *S. salina* biomass to solvent ratio (A), duty cycle (B), and amplitude (C) (n = 3).

	Experimental Factors		
Runs	A: Biomass to Solvent Ratio (mg·mL^−1^)	B: Duty Cycle (%)	C: Amplitude (%)
1	1.5	40	70
2	1.5	70	40
3	1.5	70	100
4	1.5	100	70
5	5	40	100
6	5	100	40
7	5	40	40
8	5	70	70
9	5	100	100
10	8.5	40	70
11	8.5	70	40
12	8.5	70	100
13	8.5	100	70

**Table 2:** Statistical significance of Box–Behnken model for all parameters studied (chl *a*, total carotenoids, PC, APC, PE, total PBPs, ethanolic and water yields, and AOX capacity), and corresponding equations for predicted response.

Parameters (Objective Function)	*p*-Value	R^2^	Equation
Chl *a* (mg·g_DW_^−1^)	<0.0001	0.90	2.01 − 1.10[B/S] + 0.2847DT + 0.2617A + 0.0389[B/S]*DT − 0.0914[B/S]*A + 0.6809DT*A + 0.9328[B/S]^2^ − 0.1211DT^2^ + 0.2689A^2^
Total carotenoids (mg·g_DW_^−1^)	<0.0001	0.70	0.1888 − 0.0067[B/S] + 0.0581DT + 0.0587A + 0.0042[B/S]*DT − 0.0326 [B/S]*A + 0.0975 DT*A + 0.0221[B/S]^2^ − 0.0026DT^2^ + 0.0885A^2^
PC (mg·g_DW_^−1^)	<0.0001	0.94	14.26 − 1.68[B/S] + 1.92DT + 1.32A − 0.2897[B/S]*DT − 0.9223[B/S]*A + 0.7611 DT*A − 1.15[B/S]^2^ − 3.49DT^2^ + 1.03A^2^
APC (mg·g_DW_^−1^)	<0.0001	0.84	11.87 + 3.04[B/S] − 1.79DT − 1.56A + 1.31[B/S]*DT − 5.00[B/S]*A − 2.92 DT*A + 0.7294[B/S]^2^ − 3.19DT^2^ + 0.6361A^2^
PE (mg·g_DW_^−1^)	<0.0001	0.84	2.09 − 0.1765[B/S] − 0.1766DT + 0.1335A − 0.1498[B/S]*DT − 0.6114[B/S]*A − 0.1958DT*A + 0.1098[B/S]^2^ − 0.6245DT^2^ + 0.4885A^2^
T_PBP_ (mg·g_DW_^−1^)	<0.0001	0.86	25.81 − 3.92[B/S] + 0.0590DT − 0.910A + 0.7327[B/S]*DT − 5.04[B/S]*A − 2.18DT*A + 0.8581[B/S]^2^ − 5.93DT^2^ + 3.15A^2^
Yield-EtOH (%_DW_)	<0.0001	0.91	23.41 − 19.28[B/S] + 1.56DT + 0.0758A − 2.74[B/S]*DT + 0.9497[B/S]*A − 1.88DT*A + 13.64[B/S]^2^ − 3.01DT^2^ + 5.71A^2^
Yield-Water (%_DW_)	<0.0001	0.96	35 + 11.72[B/S] − 3.21DT − 2.73A + 4.19[B/S]*DT − 1.97[B/S]*A − 2.59DT*A − 8.15[B/S]^2^ + 5.17DT^2^ + 4.72A^2^
AOX-EtOH (mg_TE_·g_DW_^−1^)	<0.0001	0.93	2.29 − 1.17[B/S] + 0.2865DT + 0.4444A + 0.0184[B/S]*DT − 0.1597[B/S]*A + 0.6220DT*A + 0.9532[B/S]^2^ − 0.1468DT^2^ + 0.5602A^2^
AOX-Water (mg_TE_·g_DW_^−1^)	<0.0001	0.78	4.57 + 0.2569[B/S] − 0.1722DT + 0.2800A − 0.0152[B/S]*DT –0.2309[B/S]*A + 0.8906DT*A + 0.7665[B/S]^2^ − 0.67937DT^2^ − 0.4600A^2^

B/S—biomass to solvent ratio; DT—duty cycle; A—Amplitude; PC—phycocyanin; APC—allophycocyanin; PE—phycoerythrin; T_PBP_—total phycobiliproteins; Yield-EtOH—yield of ethanolic extract; Yield-Water—yield of the water extract; AOX-EtOH—antioxidant capacity of ethanolic extract; AOX-Water—antioxidant capacity of water extract.

**Table 3:** Experimental results pertaining the design of Box–Behnken model for all variables evaluated (chl *a*, total carotenoids, PC, APC, PE, total PBPs, ethanolic and water yields, and AOX capacity. Bold style highlights the best experimental value obtained by each studied parameter.

Experimental Factors			Variables Evaluated									
B/S (mg·mL^−1^)	Duty Cycle (%)	Amplitude (%)	Chl *a* (mg·g_DW_^−1^)	Total Carotenoids (mg·g_DW_^−1^)	PC (mg·g_DW_^−1^)	APC (mg·g_DW_^−1^)	PE (mg·g_DW_^−1^)	T_PBP_ (mg·g_DW_^−1^)	Yield-EtOH (%_DW_)	Yield-Water (%_DW_)	AOX-EtOH (mg_TE_·g_DW_^−1^)	AOX-Water (mg_TE_·g_DW_^−1^)
1.5	40	70	4.27 ± 0.13	0.21 ± 0.04	6.26 ± 0.27	5.99 ± 0.25	1.47 ± 0.08	13.74 ± 1.53	47.65 ± 9.16	24.59 ± 1.30	4.47 ± 0.26	3.84 ± 0.22
1.5	70	40	3.61 ± 0.25	0.26 ± 0.07	9.90 ± 0.20	9.60 ± 0.67	2.30 ± 0.00	23.62 ± 0.97	**69.71 ± 0.00**	22.24 ± 0.28	4.35 ± 0.10	4.43 ± 0.02
1.5	70	100	**4.46 ± 0.21**	0.32 ± 0.01	14.47 ± 0.32	15.24 ± 0.16	**3.98 ± 0.07**	32.82 ± 1.33	62.97 ± 1.55	18.77 ± 0.90	**5.27 ± 0.10**	5.00 ± 0.11
1.5	100	70	4.13 ± 0.04	0.31 ± 0.00	10.93 ± 0.06	2.31 ± 0.01	1.46 ± 0.19	15.24 ± 1.18	50.34 ± 3.10	14.68 ± 0.31	4.39 ± 0.06	4.50 ± 0.79
5	40	100	1.07 ± 0.05	0.17 ± 0.01	10.11 ± 0.11	14.36 ± 0.03	2.38 ± 0.01	26.65 ± 0.02	22.28 ± 1.84	**51.37 ± 0.72**	2.08 ± 0.07	1.54 ± 0.04
5	100	40	1.89 ± 0.10	0.19 ± 0.01	11.95 ± 0.49	10.12 ± 0.29	1.91 ± 0.05	23.80 ± 0.50	26.17 ± 1.01	43.57 ± 1.45	2.09 ± 0.11	3.94 ± 0.02
5	40	40	2.05 ± 0.01	0.17 ± 0.01	9.79 ± 0.54	10.39 ± 0.20	1.92 ± 0.08	22.16 ± 0.27	20.90 ± 1.67	49.69 ± 0.45	2.13 ± 0.06	4.47 ± 0.12
5	70	70	2.05 ± 0.07	0.19 ± 0.00	14.26 ± 0.33	11.87± 0.51	2.09 ± 0.23	25.81 ± 0.28	23.41 ± 0.42	35.00 ± 0.25	2.29 ± 0.13	4.57 ± 0.08
5	100	100	3.63 ± 0.17	**0.57 ± 0.06**	15.31 ± 0.47	2.42 ± 0.10	1.59 ± 0.07	19.55 ± 0.67	35.06 ± 0.85	34.90 ± 0.82	4.50 ± 0.06	4.18 ± 0.05
8.5	40	70	1.44 ± 0.03	0.16 ± 0.00	8.95 ± 0.01	13.91 ± 0.49	1.98 ± 0.06	24.78 ± 0.82	23.20 ± 0.39	4.98 ± 0.63	1.77 ± 0.03	4.85 ± 0.15
8.5	70	40	2.15 ± 0.03	0.36 ± 0.04	15.64 ± 0.78	**21.23 ± 0.88**	2.61 ± 0.29	**36.90 ± 1.12**	20.63 ± 1.05	48.30 ± 0.40	2.66 ± 0.18	5.09 ± 0.15
8.5	70	100	2.63 ± 0.04	0.32 ± 0.01	**17.22 ± 0.54**	6.87 ± 0.31	1.84 ± 0.15	25.94 ± 0.39	17.70 ± 0.72	36.95 ± 0.84	2.93 ± 0.08	4.89 ± 0.12
8.5	100	70	1.45 ± 0.05	0.16 ± 0.00	12.38 ± 0.02	15.45 ± 0.15	1.37 ± 0.15	29.21 ± 0.31	14.92 ± 1.35	47.83 ± 0.71	1.75 ± 0.02	**5.28 ± 0.07**

B/S—biomass to solvent ratio; DT—duty cycle; A—Amplitude; PC—phycocyanin; APC—allophycocyanin; PE—phycoerythrin; T_PBP—_total phycobiliproteins; Yield-EtOH—yield of ethanolic extract; Yield-Water—yield of the water extract; AOX-EtOH—antioxidant capacity of ethanolic extract; AOX-Water—antioxidant capacity of water extract.

**Table 4:** Best conditions for B/S ratio, duty cycle, and amplitude, leading to maxima of objective functions (PC, APC, PE, total PBPs, chl *a*, total carotenoids, yields, and AOX capacity) for both water and ethanolic extracts, and corresponding desirability.

	Model Prediction				
Analyzed Objective Functions	B/S Ratio (mg·mL^−1^)	Duty Cycle (%)	Amplitude (%)	Max. Predicted Value	Desirability
**Water extracts**					
PC (mg·g_DW_^−1^)	6	80	100	17.21 ± 0.86	0.942
APC (mg·g_DW_^−1^)	8.5	70	40	22.82 ± 2.52	1
PE (mg·g_DW_^−1^)	1.5	60	100	3.61 ± 0.32	1
Total PBPs (mg·g_DW_^−1^)	8.5	70	40	38.97 ± 2.74	1
Yield-water (%_DW_)	8.3	100	40	54.96 ± 2.72	1
AOX-water (mg_TE_·g_DW_^−1^)	8.2	70	70	5.44 ± 0.05	1
**Ethanolic extracts**					
Chl *a* (mg·g_DW_^−1^)	1.5	100	100	5.47 ± 0.42	1
Total carotenoids (mg·g_DW_^−1^)	1.5	100	100	0.57 ± 0.06	1
Yield-EtOH (%_DW_)	1.5	100	100	64.32 ± 6.31	0.905
AOX-EtOH (mg_TE·_g_DW_^−1^)	1.5	100	100	6.32 ± 0.38	1

PC—phycocyanin; APC—allophycocyanin; PE—phycoerythrin; T_PBP—_total phycobiliproteins; Yield-EtOH—yield of ethanolic extract; Yield-Water—yield of the water extract; AOX-EtOH—antioxidant capacity of ethanolic extract; AOX-Water—antioxidant capacity of water extract.

**Table 5:** Comparison of ultrasound-assisted extraction maximum predicted values by the model with classical and *Precellys*-based extractions, performed under the best conditions in terms of B/S ratio, for phycoyanin extraction. Different subscript letters mean *p* < 0.05 (in a row).

Parameters	UAE (max. Predicted)	Classical	*Precellys*
**Aqueous extracts**			
PC (mg·g_DW_^−1^)	**17.21 ± 0.86 ^a^**	15.30 ± 1.51 ^a^	11.64 ± 0.08 ^b^
APC (mg·g_DW_^−1^)	8.65 ± 2.52 ^a^	**18.15 ± 0.72 ^b^**	4.36 ± 0.45 ^c^
PE (mg·g_DW_^−1^)	2.28 ± 0.32 ^a^	**7.40 ± 0.46 ^b^**	2.57 ± 0.13 ^a^
Total PBPs (mg·g_DW_^−1^)	27.23 ± 2.74 ^a^	**38.56 ± 2.96 ^b^**	20.02 ± 1.81 ^c^
Yield-water (%_DW_)	**38.15 ± 2.72 ^a^**	27.15 ± 0.74 ^b^	36.31 ± 0.43 ^a^
AOX-water (mg_TE_·g_DW_^−1^)	**4.62 ± 0.55 ^a^**	4.32 ± 0.32 ^a^	1.81 ± 0.07 ^b^
**Ethanolic extracts**			
Chl *a* (mg·g_DW_^−1^)	2.60 ± 0.42 ^a^	**4.75 ± 0.21 ^b^**	3.27 ± 0.18 ^a^
Total carotenoids (mg·g_DW_^−1^)	**0.32 ± 0.06 ^a^**	0.23 ± 0.01 ^a^	0.18 ± 0.01 ^a, b^
Yield-EtOH (%_DW_)	25.61 ± 6.31 ^a^	**43.27 ± 5.14 ^b^**	42.48 ± 0.85 ^b^
AOX-EtOH (mg_TE_·g_DW_^−1^)	**3.28 ± 0.38 ^a^**	2.92 ± 0.22 ^a^	0.33 ± 0.01 ^b^

## Data Availability

The authors confirm that the data supporting the findings of this study are available within the article.
